# Vitrimerization: A Novel Concept to Reprocess and Recycle Thermoset Waste via Dynamic Chemistry

**DOI:** 10.1002/gch2.201800076

**Published:** 2019-04-07

**Authors:** Liang Yue, Vahab Solouki Bonab, Dian Yuan, Ammar Patel, Vahid Karimkhani, Ica Manas‐Zloczower

**Affiliations:** ^1^ Department of Macromolecular Science and Engineering Case Western Reserve University Cleveland OH 44106 USA; ^2^ Singular Genomics Systems Inc. 10931 N. Torrey Pines Road La Jolla CA 92037 USA

**Keywords:** dynamic bonding, nanocomposites, recycling, thermoset waste, vitrimers

## Abstract

A new approach for reprocessing of existing thermoset waste is presented. This work demonstrates that unrecyclable thermoset materials can be reprocessed using the concept of associative dynamic bonding, vitrimers. The developed recycling methodology relies on swelling the thermoset network into a solution of a catalyst, which enables transesterification reactions allowing dynamic bond exchange between ester and hydroxyl groups within the thermoset network. Thermal and mechanical properties for recycled polyurethane and epoxy networks are studied and a strategy to maintain the properties of recycled materials is discussed. The developed methodology promises recycling and even upcycling and reprocessing of previously thought intractable materials. Moreover, processability of vitrimerized thermosets with common thermoplastic manufacturing methods opens up the possibility of tuning recycled networks by adding nanoparticles. This flexibility keeps the application window of recycled thermosets very broad.

Classic thermosets unlike thermoplastics are chemically crosslinked permanent polymer networks that cannot be dissolved in any solvents or melt.^1^ These networks can exhibit a glass transition temperature (*T*_g_) lower than the designed application service temperature (elastomer/rubber) or higher (thermoset resins). Usually, thermoset resins exhibit significant benefits in comparison with thermoplastics in terms of high mechanical properties, dimensional stability, and high thermal/creep/and chemical resistance. These outstanding properties in combination with their light weight make thermoset resins in their pure or fiber reinforced composites forms ideal candidates for find applications in many important and high‐tech areas such as automotive industry, adhesives, coatings, shore structures, clean energy production, solar cell encapsulations, electronic packaging, and so on.^2^, ^3^ Traditionally, the golden rule of material design was to have polymers that maintain structural strength, thermal and electrical resistance characteristics even after prolonged use. In this view, thermoset materials are ideal candidates for many common and engineering applications. However, their main advantage, which is retaining a lasting and intractable 3D structure, creates also a crucial disadvantage since they cannot be recycled or reprocessed. Environmental issues regarding very long degradation time of polymers is one of the biggest concerns of new material design and development.^4^, ^5^, ^6^, ^7^, ^8^ To understand better how big is this problem, it should be mentioned that between 2010 and 2015, annually more than 10 million tons of thermosets produced that was around 18% of total polymer products. Recently, the European Commission set a target of 2030 to increase recycling of plastic packaging stating that “the only long term solution is to reduce plastic waste by recycling and reusing more.”^9^ This applies for both thermoplastics and thermosets.

Due to the importance of polymer recycling, scientists are interested in the concept of designing materials based on a cradle‐to‐cradle life cycle or “design to degrade.”^6^, ^8^, ^10^, ^11^, ^12^, ^13^, ^14^, ^15^ A very interesting strategy to induce reformability and healing in chemically crosslinked polymer networks is by using exchangeable chemical bonds that will lead to a dynamic crosslinked network.^16^, ^17^, ^18^, ^19^, ^20^, ^21^, ^22^, ^23^, ^24^, ^25^, ^26^, ^27^, ^28^, ^29^, ^30^, ^31^, ^32^, ^33^, ^34^, ^35^, ^36^, ^37^, ^38^, ^39^, ^40^, ^41^ Polymeric systems containing such exchangeable bonds are covalent adaptable networks (CANs).^16^ CANs are classified into two different categories based on their exchange mechanisms. The first category comprises networks where the exchange mechanism of crosslinks is dissociative, which most of the crosslinks break under certain conditions (temperature,^37^, ^39^ UV‐light exposure,^22^ pH,^17^, ^33^) and with a change in the conditions they re‐form again. Dissociative CANs demonstrate a sudden and significant decrease in the viscosity with breaking of the crosslinking bonds.^42^ In the second category, the mechanism of crosslinking is associative and a crosslinking bond does not break until a new bond forms, which makes the network permanent and dynamic.^24^, ^25^, ^26^, ^27^, ^35^, ^41^

Vitrimers^21^, ^24^, ^28^, ^29^, ^30^, ^32^, ^35^, ^41^, ^42^, ^43^, ^44^, ^45^, ^46^, ^47^, ^48^, ^49^ belong to the second category of CANs in which the crosslinking bonds have an associative nature resulting in the ability of the material to change its topology via exchange reactions. These reactions normally are triggered by heat, which causes the gradual decrease in system viscosity with increasing temperature and provides malleability to the network. This allows the vitrimer thermoset to be reprocessed using conventional thermoplastic processing techniques such as extrusion, injection and compression molding, 3D printing, etc., an unprecedented advantage solely afforded to thermoplastics up until now. The viscosity of vitrimers is governed by the chemical exchange reaction at elevated temperatures and like vitreous silica and unlike dissociative networks and thermoplastics, decreases gradually.^50^ Vitrimers maintain permanent networks at all temperatures until degradation, and they can swell but not dissolve in specific solvents. However, swelling ratios are higher for these networks in comparison with the nondynamic ones. In recent years, different CANs are reported based on rich library of dynamic covalent chemistry like Diels–Alder reaction, transesterifications, sulfur chemistries, olefin metathesis, alkoxyamine, imine and vinylogous urethane exchange, urea‐urethane exchanges, siloxane exchanges, bronic ester exchanges, Shiff base, dioxaborolane metathesis, and so on.^51^, ^52^

It must be mentioned here that while the dynamic networks and CANs rely on creating new thermosets that are recyclable and reprocessable, there is already millions of tons of waste thermosets, or thermoset parts in use that are not designed to be degradable.^6^, ^15^

Currently, there are three main approaches for recycling thermoset waste or thermoset waste based composites: (i) thermal, (ii) chemical, and (iii) mechanical.^53^, ^54^, ^55^ The thermal method is mainly for energy recovery and in some cases filler recovery, too.^56^, ^57^, ^58^ The chemical method is based on degradation or depolymerization into smaller molecules.^59^, ^60^ In the mechanical method, waste thermosets break down to small particles through the application of energy.^61^, ^62^ Later, these particles can be used as filler in thermoset or thermoplastic matrices. However, to the best of our knowledge, there is no way to use waste thermosets directly to make new parts as is the case with thermoplastic materials.

Here, we propose a method to recycle thermoset waste. The developed process coined as vitrimerization involves changing the permanent networks into dynamic ones with the use of an appropriate catalyst solution which will turn them into vitrimers. Thermosets will be swollen in a solution of an appropriate catalyst to allow the catalyst to diffuse into the network. Upon sufficient catalyst infusion, the solvent can be removed to be recycled for the next batch of thermosets to be vitrimerized. The catalyst left behind in the thermoset will facilitate the occurrence of exchange reactions at elevated temperature rendering the system a dynamic network. Thus, the thermoset becomes recyclable and healable for many times. This is especially important since the maximum application temperature for CANs is limited by the temperature at which they are designed to be dynamic, not by their degradation temperature. Hence, this design dilemma restricts the CANs application range by comparison with classic thermosets suitable for many high temperature usages. Our method affords a thermoset to be used for its intended application after which it can be converted into a vitrimer to serve a secondary purpose. Additionally, we show here that processability of vitrimerized thermosets gives the opportunity of using nanoparticles to tune their properties and even upcycle them. It is worthy of note that we have just implemented transesterification chemistry here, but the concept can be extended to other alternative chemistry methodologies.

The transesterification reaction^26^, ^28^, ^29^, ^35^, ^44^, ^46^, ^63^, ^64^ was selected as a model chemistry for vitrimerization in this research because of its importance and abundance in many thermoset systems. Any thermoset material that has ester groups and freely available hydroxyl groups can be infused with a catalyst that supports transesterification and thereby be converted into a vitrimer. In this research the effect of vitrimerization on recyclability of epoxy and polyurethane as two widely used thermoset materials has been investigated.

The vitrimerization process has just two requirements: (i) a solvent that can swell the thermoset material, and (ii) a catalyst that can facilitate dynamic reactions that is either soluble or miscible in the solvent.

The catalyst employed in this work, tin (II) 2‐ethylhexanoate (Sn(Oct)_2_) was chosen for its high catalytic activity in transesterification reactions^46^, ^65^ as well as its high stability in most media.^66^ Other catalysts used for transesterification such as zinc acetylacetonate,^26^, ^35^, ^44^, ^64^ triazobicyclodecene,^29^, ^64^ triphenylphosphine,^64^ zinc acetate,^26^ and zinc octoate^67^ may also be used. Generally, the catalyst was dissolved in a suitable solvent with a catalyst concentration of 10 wt%. A concentration less than this value did not allow for enough catalyst infusion into the network resulting in poor reprocessability.

The Vitrimerization process: The infusion of catalyst into the sample to make a vitrimerized material is also dependent on temperature. The infusion was much more successful if it was carried out at a temperature above the *T*_g_ of the sample. It is believed that the solvent swelling at temperatures higher than the network's *T*_g_ facilitated the diffusion of the catalyst molecules into the thermoset network. **Figure** **1** shows a schematic of the vitrimerization process. In general, a 10 wt% catalyst solution (solvent varied based on temperature of infusion) was prepared, and small pieces of the thermoset samples (≈2 mm * 2 mm * 2 mm) were immersed into the solution for 48 h. The samples were then washed with ethanol to remove the catalyst from the surface and dried in a vacuum oven for 12–24 h. More specifically, the infusion for PU (having a *T*_g_ of ≈−7 °C) was successful at room temperature using dichloromethane (DCM) as the solvent. The samples after infusion and washing with ethanol were dried at 80 °C for 24 h. The epoxy sample which had a *T*_g_ of 95 °C was infused with the catalyst at 140 °C, since its glassy behavior at room temperature would prevent significant catalyst diffusion. Logically, a high temperature boiling point solvent dimethylformamide replaced DCM for the hard network infusion. The infusion was carried out for 48 h, following which the samples were washed with ethanol and dried at 140 °C in a vacuum oven for 24 h.

**Figure 1:**
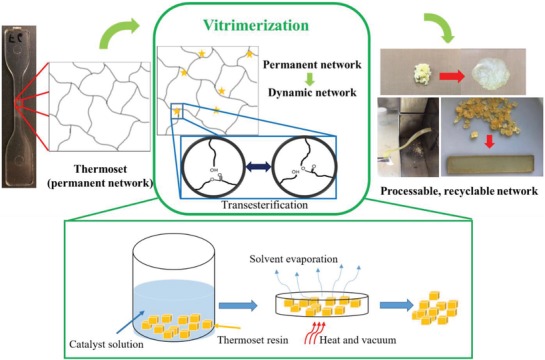
An overview of the process wherein a permanent thermoset network can be extruded, injection molded, and compression molded after vitrimerization.

Processing of the vitrimerized samples: The infused or “vitrimerized” samples were processed by compression molding and extrusion. Figure 1 shows samples made from each of these processes. In the case of compression molding, preheating time, pressing time, and temperature played a role in determining final film quality. PU was processed at 200 °C with 5 min of preheating time and 5 min of pressing at a pressure of 5 metric tons. A higher temperature resulted in the onset of degradation of the PU material. The epoxy samples were hot pressed at 250 °C with 20 min of preheating time and then pressed for 15 min at a pressure of 5 metric tons. Temperatures less than 250 °C resulted in incomplete films.

Extruded samples were obtained by feeding the vitrimerized material into the hopper of a twin screw counter rotating minilab operating at a temperature of 205 °C and at an RPM of 15. Besides extrusion, some samples in the minilab were also forced into a mold to form rectangular shaped samples as can be seen in Figure 1, a process akin to injection molding.

Properties of the vitrimerized samples: Dog bone shaped PU thin films obtained from hot pressing were compared for mechanical testing with their neat counterparts, i.e., PU samples which were not vitrimerized. Rectangular shaped samples obtained from the minilab were used for mechanical testing in the case of the epoxy system. Those properties are shown in **Figure** **2**a,b and summarized in the Supporting Information. It can be seen that after recycling, 44% of modulus, 30% of ultimate strength, and 78% of extensibility for PU could be recovered. In the case of the epoxy system, 73.5% of modulus, 63% of ultimate strength, and 82% of extensibility were recovered.

**Figure 2:**
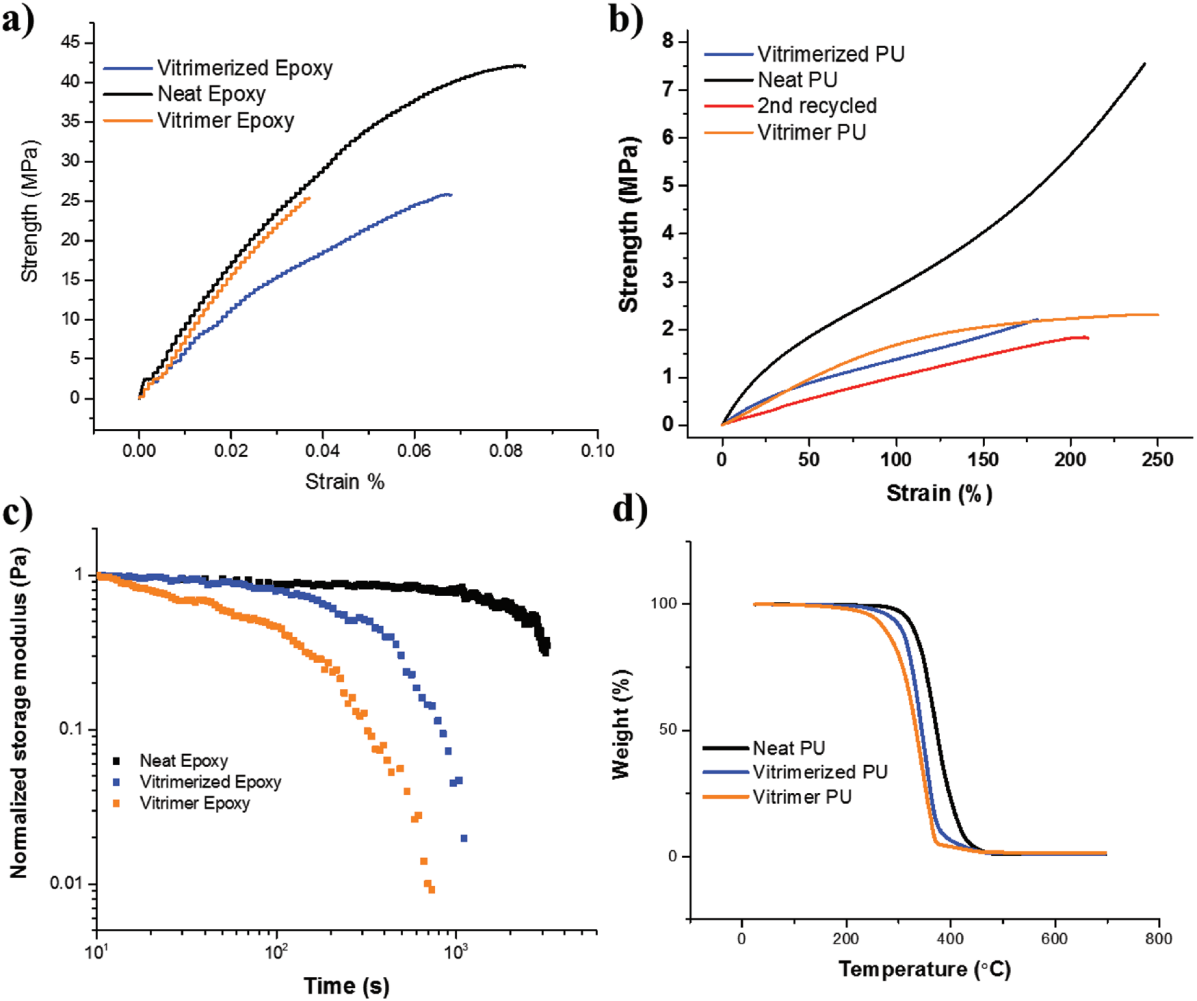
a,b) Representative tensile curves for the epoxy and PU system, respectively. b) The effect of a second recycling on the mechanical properties. c) Stress relaxation behavior for the neat, vitrimerized, and vitrimer epoxy system. d) Thermal decomposition behavior of the neat, vitrimerized, and vitrimer PU system.

The decrease in properties observed is due to the infusion of the catalyst. The presence of the catalyst has a deleterious effect on the mechanical and thermal properties of the system, i.e., a vitrimer exhibits generally lower properties than the same thermoset network with no catalyst. Rationally, the vitrimerization process by itself does not decrease the properties of the system lower than that of the vitrimer. Stress relaxation shows that we were successful in converting the unrecyclable thermoset into a vitrimer using the vitrimerization process. The neat system having no catalyst is an intractable 3D network. It was expected that it would be unable to relax. Both, the vitrimer and vitrimerized network stress relax quite significantly due to the transesterification reaction. The rate of relaxation as observed by Leibler is dependent on the amount of catalyst present in the system.^64^ Figure 2c implies that the amount of the catalyst present in the vitrimerized system is lesser than the vitrimer system since the vitrimer relaxes faster than the vitrimerized network. The amount of catalyst could not be observed directly from the TGA curve possibly because the network degrades at similar temperatures as the catalyst. However, the onset of degradation is also affected by the amount of the catalyst as can be seen in Figure 2d. The vitrimer starts to degrade at a lower temperature when compared to the vitrimerized system. Both the systems with catalyst degrade at a lower temperature when compared to the neat system. This is in line with the results obtained from the mechanical properties.

The effect of multiple recycling on vitrimerized samples was also investigated as can be seen in Figure 2b and Figure S1 (Supporting Information). Previously thought to be unrecyclable PU waste, the vitrimerized samples could now be reprocessed up to two times. The third recycling resulted in a paste unable to form a film of any substantial strength. In the 2nd recycling process there was a 10% decrease in ultimate tensile strength and a 50% decrease in modulus, while the elongation at break increased by 35% by comparison with the first recycle. The multiple recycling did not however have any effect of the thermal decomposition temperatures of the material as is observed in Figure S1 of the Supporting Information.

It must be noted here that the vitrimerization process is not completely optimized. It is expected that by controlling the amount of catalyst incorporated into the system, streamlining the infusion process, the drying and processing, the properties of the recycled material can be improved even further.^32^ Since vitrimerization also allows for the processing of a thermoset just like a thermoplastic, it affords the unique advantage of incorporating fillers into the thermoset network during recycling to compensate for the drop in mechanical properties due to the catalyst. The addition of fillers to enhance the properties of vitrimers has already been studied extensively.^43^, ^44^, ^48^, ^49^, ^63^ Just as an example, 10 wt% of highly branched carbon nanotubes with a diameter of 7–9 nm known as carbon nanostructures (CNS) were extruded along with the vitrimerized PU samples with the same parameters mentioned above and tested for mechanical properties as can be seen in **Figure** **3**c. The nanocomposite exhibited brittle behavior showing an equivalent ultimate tensile strength to that of the neat sample and a modulus that was ≈2.5 times higher. This experiment demonstrates the potential of vitrimerization to even upcylce the waste materials and tune and improve their properties for higher performance applications in comparison to their original use.

**Figure 3:**
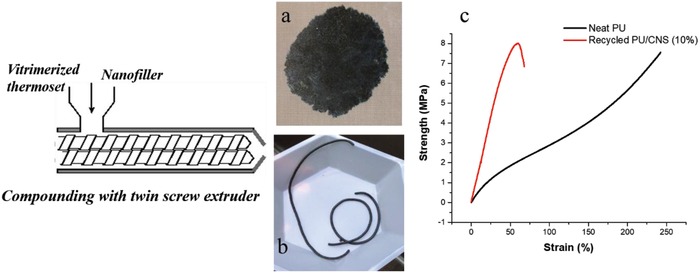
a) Vitrimerized PU with 10 wt% CNS hot‐pressed into a thin film. b) Vitrimerized PU with 10 wt% extruded into strands. c) Representative tensile curves of neat and nanofilled PU system.

In this proof of concept work, we have clearly shown that PU and epoxy thermoset materials, previously thought to be waste and a burden to the environment past their intended use can be recycled and reprocessed once more via the vitrimerization process. Since the solvent is just a facilitator for catalyst infusion, all the solvent used in the process can also be recycled. While conventional processing techniques such as compression molding, extrusion, and injection molding have been used in this work, newer processes for shaping these samples are currently being investigated for viability. Ongoing work related to optimization of the vitrimerization process in terms of temperature and catalyst concentration is expected to result in an increase of the properties of the reprocessed materials. We have further shown that during the reprocessing, incorporation of suitable fillers like CNS may significantly improve the properties to resemble or even surpass those of the original material. Albeit, in this work we have taken advantage of the transesterification chemistry for dynamic bonding, with the right catalyst or a combination of different catalysts, this work can be extended to other vitrimer chemistries.^42^

## Experimental Section

*Preparation of Neat Samples*: Crosslinked polyurethane was synthesized by polymerizing polycaprolactone triol (Mw = 900 g mol^−1^) with 1,4‐phenylene diisocyanate (PPDI). 10 g polyol was degassed under vacuum at 80 °C overnight and then transferred into a sealed three neck reactor under nitrogen. 40 mL of DCM was added to the reactor and stirred to dissolve the polyol. In order to have excess OH groups in PU, the required amount of PPDI was calculated to maintain the [NCO]/[OH] ratio as 0.9. The PPDI was also dissolved in 40 mL of DCM. This PPDI solution was added to the polyol solution at 40 °C and stirred vigorously for 3 min. The solution was cast on a Teflon plate and the solvent was left to evaporate under nitrogen flow at room temperature for 48 h. The resulting films were placed in a vacuum oven at 80 °C for 24 h to remove any trace of solvent from the PU. A TGA temperature ramp confirmed that no solvent remained in the system.

Epoxy networks were synthesized using diglycidyl ether of bisphenol A (DGEBA) and glutaric anhydride. The reactants were hand mixed together using a glass rod such that the ratio of [epoxy groups]:[anhydride groups] is 1:1. An imidazole catalyst was added at 1 wt% with respect to DGEBA to accelerate the reaction. The mixture was poured into a mold and cured at 80 °C and then 160 °C for 8 h each.

*Tensile Testing*: Uniaxial tensile tests for PU were carried out at room temperature on a Zwick/Roell (Model Z0.5) with load cell of 100N on at least four samples at an extension rate of 10 mm min^−1^. Samples had dumbbell shape and were cut from films prepared by compression molding. Tensile tests for the epoxy system were conducted on a Zwick/Roell with a load cell of 500N at an extension rate of 1 mm min^−1^. Samples were rectangular shaped and were obtained from the minilab in a process that will be explained later in this paper.

*TGA*: Thermal decomposition tests were run on samples on a Q500 TA instruments machine under N2 flow. Samples were ramped up to 700 °C at 10 °C min^−1^.

*Stress Relaxation*: The Ares G2 Rheometer was used to measure the stress relaxation behavior of all samples. Samples were studied in parallel plate geometry at a temperature of 200 °C. Samples were subjected to a strain percentage following which the relaxation in storage modulus was observed with time. Care was taken to ensure that the strain values were within the viscoelastic regimes of all samples.

## Conflict of Interest

The authors declare no conflict of interest.

## Supporting information

Supplementary
