# Supplementary material for: Vitrimerization: A Novel Concept to Reprocess and Recycle Thermoset Waste via Dynamic Chemistry
Source: Glob Chall. 2019 Apr 7;3(7):1800076. doi: 10.1002/gch2.201800076 (PMC6607417; doi:10.1002/gch2.201800076)

Copyright WILEY-VCH Verlag GmbH & Co. KGaA, 69469 Weinheim, Germany, 2019.

Global Challenges
Open Access

Supporting Information

for *Global Challenges,* DOI: 10.1002/gch2.201800076

Vitrimerization: A Novel Concept to Reprocess and Recycle Thermoset Waste via Dynamic Chemistry

*Liang Yue, Vahab Solouki Bonab, Dian Yuan, Ammar Patel, Vahid Karimkhani, and Ica Manas-Zloczower**

Supporting Information

Table Sa) Mechanical properties comparing neat, vitrimer and vitrimerized samples for PU system

Samples	Strength (MPa)	Modulus (MPa)	Elongation at break (%)
Neat	7.84±0.85	5.56±0.46	246.0±37.1
Vitrimerized 1st recycle	2.35±0.22	2.43±0.06	192.0±16.9
Vitrimer 1 wt%	2.460±0.277	2.41±0.16	195±19
Vitrimer 5 wt%	2.370±0.107	1.791±0.100	261±40
Vitrimer 10 wt%	3.227±0.223	2.481±0.063	241±14

Table Sb) Mechanical properties comparing neat, vitrimer and vitrimerized samples for epoxy system

Samples	Strength (MPa)	Modulus (MPa)	Elongation at break (%)
Neat	40.00±1.41	802.00±100.41	8.0±0.1
Vitrimerized 1st recycle	26.00	600	6.8
Vitrimer 5wt%	22.13±5.20	918.00±11.16	3.80±0.03

Table Sc) Mechanical properties of a PU system with multiple recycling and addition of nanofillers

Samples	Strength (MPa)	Modulus (MPa)	Elongation at break (%)
Neat	7.84±0.84	5.55±0.45	246.0±37.1
Recycled (1st)	2.35±0.22	2.43±0.06	192.0±16.9
Recycled (2nd)	2.10±0.22	1.22±0.22	259±52
Recycled 10 wt.% CNS	7.251±1.532	13.48±2.88	67.5±28.6

Figure S1: Effect of multiple recycling on the thermal properties of epoxy system

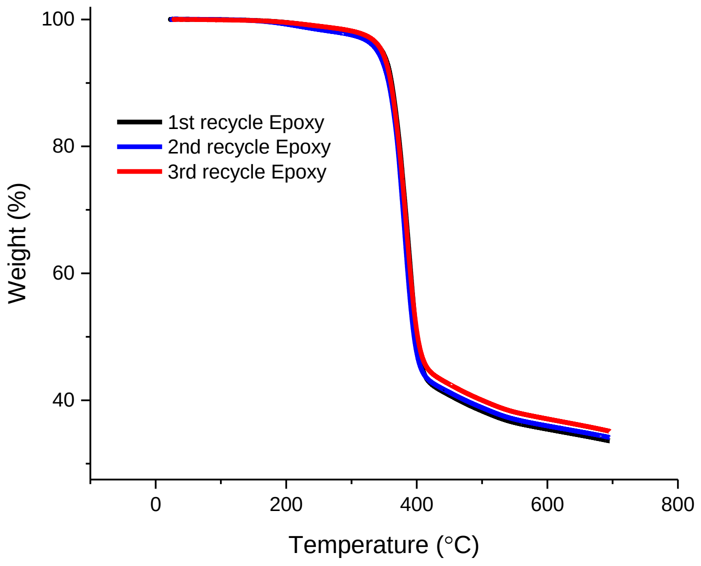

Supplement: Supplementary file 1 — Supplementary [file GCH2-3-1800076-s001.pdf]
